# Unusual Behavior of *Xylosandrus compactus* (Coleoptera: Scolytinae) on Carob Trees in a Mediterranean Environment

**DOI:** 10.3390/insects10030082

**Published:** 2019-03-23

**Authors:** Antonio Gugliuzzo, Giulio Criscione, Giovanna Tropea Garzia

**Affiliations:** Department of Agriculture, Food and Environment, University of Catania, Via Santa Sofia 100, 95123 Catania, Italy; giulio.criscione.93@gmail.com (G.C.); gtgarzia@unict.it (G.T.G.)

**Keywords:** ambrosia beetles, black twig borer, *Ceratonia siliqua*, invasive pest, new signs

## Abstract

*Xylosandrus compactus* (Eichhoff), commonly known as the black twig borer, was reported in Sicily (Italy) at the end of 2016, infesting Carob tree (*Ceratonia siliqua* L.) twigs, large branches, and trunks. Previous research indicated that *X. compactus* attacks only small twigs and branches, not large branches and tree trunks. This unusual behavior was monitored through the two following years in five sites in Ragusa province (Sicily, Italy). For each of the monitored sites, the diameter of the infested trunks and branches was recorded. Samples of branches and trunks presenting galleries were removed from the trees and analyzed in the laboratory. *Xylosandrus compactus* occurred on branches of all monitored trees, while the percentage of infested trunks of carob trees ranged from 60% to 80%. Inside the infested galleries, all biological stages of *X. compactus* were found. Infestations were also recorded on trunks and branches with diameters greater than 80 cm and 30 cm, respectively. The mean number of *X. compactus* specimens inside the galleries was recorded and gallery shape was described. Unordinary behaviors, like the one described here for the first time, can affect the current efficiency of management recommendations.

## 1. Introduction

Ambrosia beetles are known as destructive pests of numerous plant hosts growing in managed and unmanaged environments. The damage is caused by the wood-boring activity of the pest. After boring into the wood, ambrosia beetles inoculate the walls of their gallery with symbiotic fungi which serves as a food source for adults and larvae. Visible symptoms of ambrosia beetle damage include leaf and stem necrosis, and flagging and wilting of twigs. In extreme circumstances, tree death can occur. Depending on the ambrosia beetle species, attacks may occur on shoots and twigs, small or large branches, the main trunk, or even the tree base [1,2,3,4,5]. After drilling, the female excavates tunnels and pushes out typical compact cylinders of frass.

The only previously documented ambrosia beetle species found damaging branches and trunks of Carob tree (*Ceratonia siliqua* L.) was *Xylosandrus crassiusculus* (Motschulsky) (Coleoptera: Scolytinae) [2,6,7]. This beetle colonizes all the woody parts of the trees, from small- to medium-diameter (2–30 cm) twigs, branches, and trunks. It is capable of attacking rather small woody parts (Ø 2.5–8 cm) but larger logs can also be damaged (e.g., up to 30 cm diameter) [7].

The exotic black twig borer, *X. compactus* (Eichhoff) (Coleoptera: Scolytinae), is a polyphagous pest found in Italy on laurel and other ornamental plants, as well as on several trees, including *C. siliqua* [8]. In Sicily, the pest was discovered in 2016 [9], but infested Carob trees did not show the signature frass cylinders protruding from the woody organs. However, these trees showed heavy infestations on large branches and trunks, visible due to the presence of several entry holes, wood necrosis, and sap emission. This behavior, studied here for the first time, is very unusual for *X. compactus*, which mainly infests small twigs.

A few studies have reported successful attacks of this beetle in small flowering dogwood (*Cornus florida* L.) branches [10] and avocado branches [11] with a maximum diameter of 2.2 cm and 5.2 cm, respectively. Other studies have reported unsuccessful boring attempts in avocado branches and trunks larger than 1.5 cm and 20 cm in diameter, respectively [12]. The plant parts mainly infested by the black twig borer are usually small twigs and lateral small branches with an outside diameter <7 mm [10,13]. In response to *X. compactus* attacks, some plants like avocado and *Croton reflexifolius* Kunth can exude sap from the entry hole as a repellent factor [12]. However, in avocado trees, the sap has been observed in successful and unsuccessful attacks, while in *C. reflexifolius* no sap was exuded when successful boring occurred. Similar observations have been made in *Acacia koa* Gray seedlings on stems larger than 10 mm in diameter [12], while no sap production was observed on attacked southern magnolia trees (*Magnolia grandiflora* L.) [14]. This suggests that not all host plants are able to produce sap as a repellent factor. In this way, the success of attacks can be facilitated by the lower quantity of sap generated by the trees under stressed conditions. Another specific type of tree defense, in response to Scolytinae attacks, is the gum exudate produced by some plants, as in the case of *X. compactus* damage on *Limonia acidissima* L. [15].

Considering that the introduction of nonnative species can sometimes result in devastating ecological and economic losses, the study of the unusual bio-ethological behavior of *X. compactus* on this new host is essential in order to develop an integrated pest management in this newly colonized environment.

## 2. Materials and Methods

The unusual behavior for *X. compactus* was studied in 2017 and 2018 in order to detect colonies inside the branches and trunks and to describe the galleries’ shapes and sizes. Field and laboratory observations were made in order to document the typical signs and symptoms of the attack on these woody parts. 

Sampling was carried out in five sites in Ragusa province. The sites chosen for sampling were recently infested by the beetle and represented different types of environments (semi-urban and natural) and altitudes (from 6 to 290 m a.s.l.). The first two sites (with 10 Carob trees per site) monitored during 2017 were located in a natural environment 1.6 km away from each other, inside the area where the pest was first observed. The other three sites, with 10 trees per site, were monitored during 2018 in new spreading areas of *X. compactus*, near the borders of the currently colonized territory.

The sites sampled in 2017 were located in Scicli (Ragusa), the first (site A) at 199 m a.s.l. and the other (site B) at 156 m a.s.l. The sites monitored in 2018 (and named C, D, and E, respectively) were located in Donnalucata (Ragusa) at 18 m a.s.l., Marina di Modica (Modica) at 6 m a.s.l., and in Donnafugata (Ragusa) at 290 m a.s.l.. The C and D sites were characterized by semi-urban environments, and the E site by a natural environment. From the beginning of sampling, all trees showed the presence of the beetle inside small twigs, tree organs typically infested by this ambrosia beetle.

For each monitored site, the diameter of the infested trunks and branches, at the level of the first entry hole from the base, was recorded. Smaller infested branches (Ø < 15 cm) were randomly sampled from the canopy of the monitored trees, cut to various sizes, and transferred to the laboratory. These were dissected the same day, at ambient room conditions, to document the gallery shape and to record the different developmental stages of the beetle present in each gallery. For large branches (Ø from 15 to 40 cm) and trunks (Ø > 30 cm), wooden layers containing the infested galleries were removed from the trees and carried into the laboratory. Specimens found inside each brood chamber were identified morphologically [16]. The shape of the galleries infested by *X. compactus* and the number of adults/gallery were recorded.

Datasets were first tested for normality and homogeneity of variance using Kolmogorov–Smirnov D test and Cochran’s test, respectively. No data transformation was needed. All of the data were analyzed with SPSS Statistics 22.0 software (IBM Corp., Armonk, NY, USA). Data analysis was performed with a factorial ANOVA (*p* < 0.05), using the organ, the year, the environment and the site as factors, and the percentage of infested trees on different woody parts as dependent variable. Mean number of adults/gallery was analyzed by means of one-way ANOVA (*p* < 0.05).

## 3. Results

**Figure 1:**
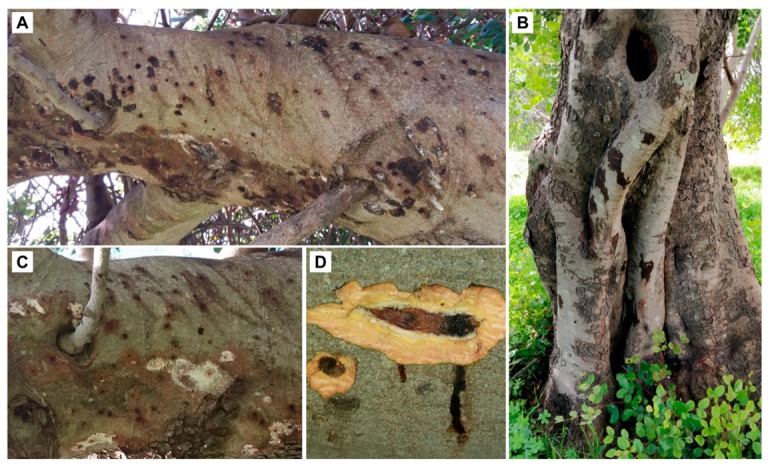
*Xylosandrus compactus* infestation on Carob tree. Heavily infested large (diameter > 30 cm) branch (**A**); trunk of a secular Carob tree (diameter at the level of first entry hole from the ground = 65 cm) with visible sap emission (**B**); entry holes with visible sap exudate associated with the formation of calcium oxalate on a large branch (**C**); and underlying necrotic tissues (**D**).

During the sampling period, all monitored plants showed the presence of *X. compactus*, both on twigs and on branches (Figure 1A). The percentage of Carob trees infested on the trunk (Figure 1B) varied from 60% (sites D and E) to 80% (sites A and B). The year (*F* = 2.416; df = 1, 153; *p* = 0.122), the environment (*F* = 1.197; df = 1, 153; *p* = 0.276), and the site (*F* = 0.127; df = 3, 153; *p* = 0.944) factors did not significantly affect the percentage of trees with infestations of each of the different woody organs, however, the organ factor did (*F* = 30.654; df = 1, 153; *p* < 0.001), because 100% of branches were infested, while the beetle was found infesting from 60% to 80% of the trunks (Table 1). The mean infested diameter ranged from 17.4 cm (site C) to 21.0 cm (site A) for the branches, and from 51.1 cm (site C) to 63.3 cm (site A) for the trunks (Table 1). However, the maximum diameters of the infested trunks and branches were 85 cm and 36 cm, respectively.

A total of 912 infested galleries were found during the whole monitoring period (Table 2). All *X. compactus* biological stages were found inside the galleries of the infested trees from spring to autumn, while only adults occurred during the winter. The mean number of adults/gallery was 19.98 ± 0.19 SE, obtained from a total of 241 galleries with only adults (Table 2). There was no significant difference among the number of adults/gallery in the different sites (*F* = 0.312; df = 4, 236; *p* = 0.870), environments (*F* = 0.187; df = 1, 239; *p* = 0.665), and years (*F* = 0.001; df = 1, 239; *p* = 0.975).

**Figure 2:**
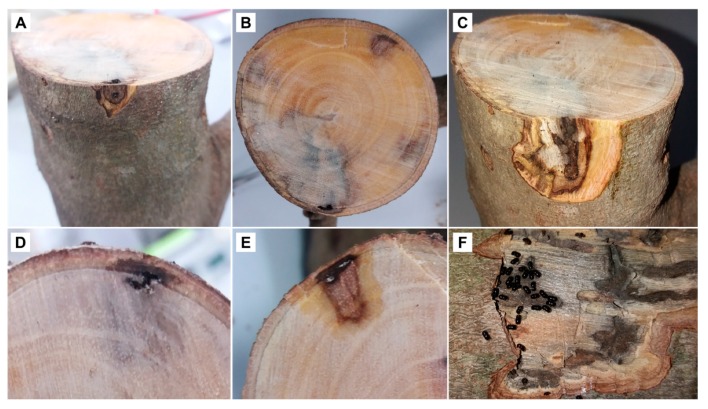
Carob tree branches infested by *Xylosandrus compactus*. Necrotic tissue below the entry hole (**A**); gallery in cross section (**B**); gallery in longitudinal section (**C**); different shapes of galleries in cross section (**D**,**E**) and in longitudinal section (**F**).

Not all attacked trees showed sap exudates outside the entry holes. Often, the sap emission from the entry holes of the big branches was associated with the formation of a white powdery material identified as calcium oxalate (Figure 1C). However, even some galleries underneath the entrance holes exuding sap showed living colonies of *X. compactus*. The wood examination beneath the entry holes of the beetle revealed a brownish staining and necrosis of the xylem caused by fungal infection (Figure 1D). Xylem necrosis was visible below the bark and extended for several centimeters from the entrance hole (Figure 2A–C). 

**Figure 3:**
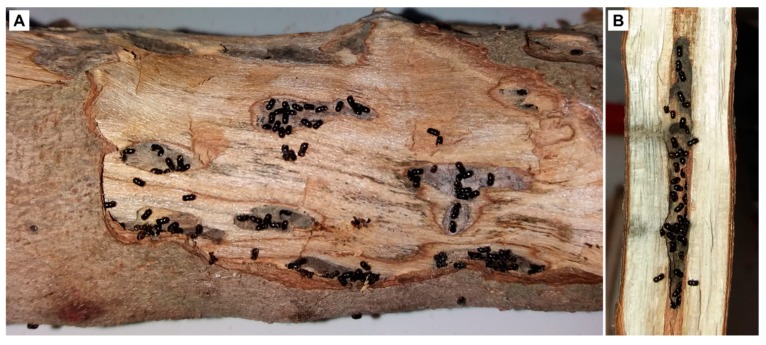
*Xylosandrus compactus* adults in several galleries of a Carob branch (**A**) and typical galleries inside a Carob twig (**B**).

Through the laboratory observations, it was possible to distinguish two kinds of galleries in branches and trunks (Figure 2D–F). The first type appeared similar to that produced by *X. compactus* in twigs, where the beetle excavates along a unique direction on either side of the initial entrance tunnel (Figure 2C). The second type of gallery, however, showed irregular shapes, and the development of the colony took place in small, ramified tunnels extending from the brood chamber (Figure 2F and Figure 3A).

## 4. Discussion

Our results showed, for the first time, that *X. compactus* successfully infested large tree branches (diameter > 20 cm) and trunks (diameter > 60 cm) of Carob trees (Figure 1B). Moreover, the same behavior was recorded in two different environments (i.e., natural vs. semi-urban), and in different years. In fact, no significant difference, in terms of percentage of infested trees according to the type of woody organ infested was found in the sites monitored in different years or in sites located in different environments. However, future observations in newly infested areas will be conducted in order to understand the causes that induced this unusual behavior on the new host plant. 

The galleries from infested branches and trunks showed irregular shapes, with small, ramified tunnels starting from the brood chamber (Figure 3A). These galleries are clearly different from those excavated out of the pith of the twigs of other host plants [13] and of the Carob tree (Figure 3B) by *X. compactus*. This behavior, related to the fact that the foundress does not reach the pith of the large woody parts, shows that the first layer of wood underlying the bark can be used by *X. compactus* and its symbionts to complete their biological cycles. The observations conducted, both in the field and in the laboratory, also showed that the trees can react to the attack with the emission of sap from the entry holes. When the sap dried, the calcium oxalate remained surrounding the holes, as described in attacked avocado trees [12]. However, this defense mechanism only prevented attack by the beetle in some cases.

The mean number of *X. compactus* adults/gallery obtained from infested Carob trees was similar to other studies where this number ranged from 1 to 41 in coffee tree [13] and from 1 to 40 in flowering dogwood [10]. This value was very similar to that found in the carob tree twigs infested by the beetle in the same sites (personal observation). This result suggests that the development of the insect is not influenced by the type of infested wood organ.

Finally, a new approach to the management of *X. compactus* in the newly colonized environment appears necessary. In fact, knowledge of this unusual bio-ethological behavior suggests that monitoring, as well as pruning, should be conducted on different organs of the infested trees, in relation to the new signs of infestation described.

## Figures and Tables

**Table 1:** Level of infestation and mean diameter of different Carob tree organs infested by *Xylosandrus compactus* in five sites in Sicily (Italy) in 2017 (A and B) and 2018 (C, D, and E).

Site	Position	Environment	Percentage of Trees by Type of Woody Organ Infested (%)		No. of Infested Organs/10 Carob Trees		Mean Diameter of Infested Woody Organs (cm ± SE)	
			Branches	Trunk	Branches	Trunk	Branches	Trunk
**A**	36°45′41″N 14°45′11″E	Natural	100	80	27	8	21.0 ± 1.2	63.3 ± 5.9
**B**	36°44′57″N 14°45′44″E	Natural	100	80	26	8	18.9 ± 1.0	54.8 ± 3.7
**C**	36°45′45″N 14°38′40″E	Semi-urban	100	70	21	7	17.4 ± 1.3	51.1 ± 6.1
**D**	36°42′40″N 14°46′22″E	Semi-urban	100	60	22	6	19.7 ± 1.3	57.7 ± 7.1
**E**	36°52′59″N 14°33′34″E	Natural	100	60	21	6	19.2 ± 1.2	61.2 ± 5.3

**Table 2:** Total number of infested galleries and mean number of *Xylosandrus compactus* adults/gallery (obtained from galleries with only adults) on different Carob tree organs in five sites in Sicily, Italy.

Year	Site	Infested Tree Organs								
		Branches (Ø < 15 cm)			Branches (Ø 15–40 cm)			Trunk		
		Infested Galleries (no.)	Galleries with Only Adults (no.)	Adults/Gallery (mean ± SE)	Infested Galleries (no.)	Galleries with Only Adults (no.)	Adults/Gallery (mean ± SE)	Infested Galleries (no.)	Galleries with Only Adults (no.)	Adults/Gallery (mean ± SE)
**2017**	A	69	17	20.65 ± 0.76	82	25	18.84 ± 0.60	46	12	20.42 ± 0.74
	B	65	16	20.38 ± 0.75	74	22	19.86 ± 0.47	49	18	20.44 ± 0.90
**2018**	C	68	17	19.41 ± 0.70	79	18	20.44 ± 0.63	42	12	20.33 ± 0.84
	D	78	19	18.95 ± 0.84	59	15	20.47 ± 0.64	37	9	19.89 ± 1.45
	E	63	16	20.31 ± 0.71	62	17	20.29 ± 0.59	39	8	19.75 ± 1.31
